# Supplementary material for: Eosinopenia as a predictor of clinical outcomes in hospitalized patients with community-acquired pneumonia: A retrospective cohort study
Source: PLoS One. 2025 Mar 6;20(3):e0314336. doi: 10.1371/journal.pone.0314336 (PMC11884692; doi:10.1371/journal.pone.0314336)
Supplement: S1 Fig — (DOCX) [file pone.0314336.s001.docx]

# **Supporting information:**

# **S1 Fig. Prognostic Accuracy of Eosinopenia and Pneumonia Severity Index in Predicting 30-day mortality**

# **S2 Fig. Prognostic Accuracy of Eosinopenia and Pneumonia Severity Index in Predicting Composite Secondary Outcomes**

# **S1 Table. Clinical Outcomes based on Eosinopenic Status in subgroup of patients with CRP**

# **S2 Table. Clinical Outcomes based on Eosinopenic Status in subgroup of patients less than 65 years old**

# **S3 Table. Mortality outcomes based on Eosinopenic Status after adjustment for steroid treatment during hospitalization**

# **S4 Table. Mortality outcomes based on Eosinopenic Status after adjustment for early (within 24 hours) steroid treatment**

**S1 Fig. Prognostic ccuracy of eosinopenia and Pneumonia Severity Index in Predicting 30-day mortality**
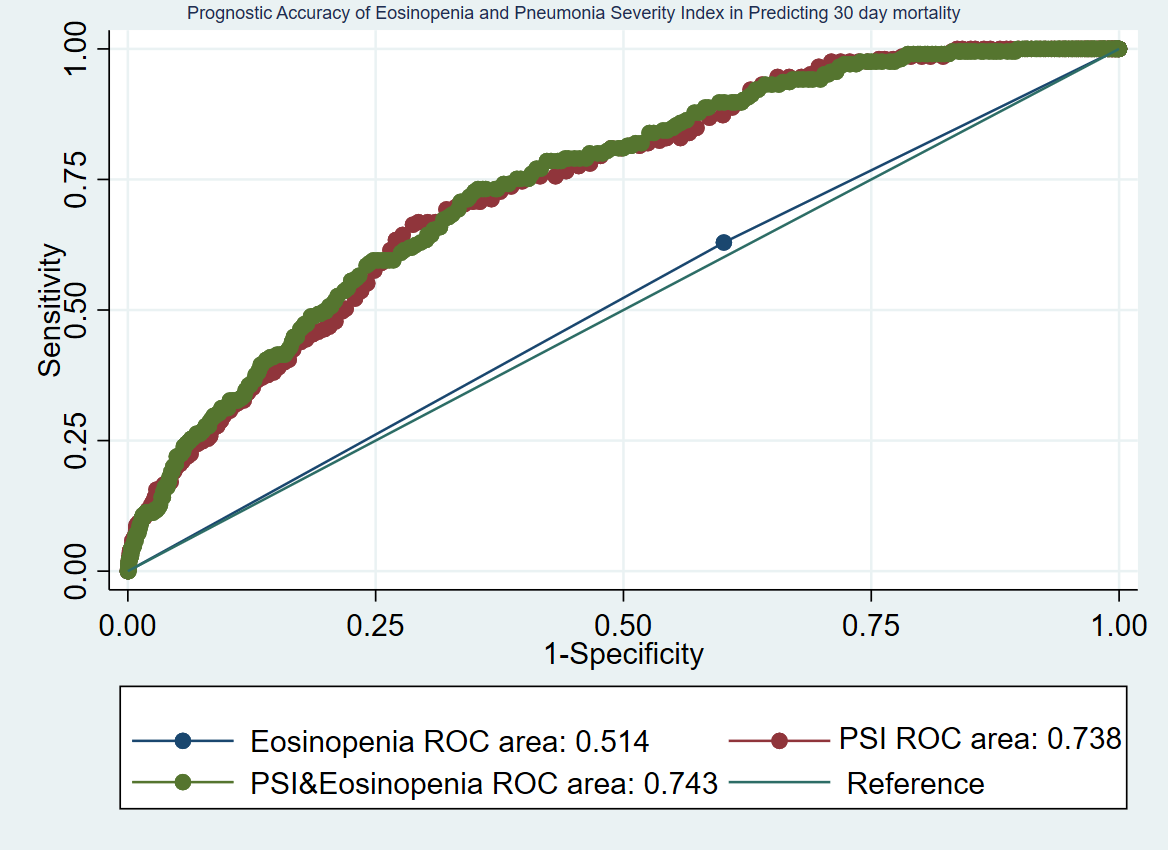


*PSI: Pneumonia severity index

**Figure S-2: Prognostic Accuracy of Eosinopenia and Pneumonia Severity Index in Predicting Composite Secondary Outcomes**


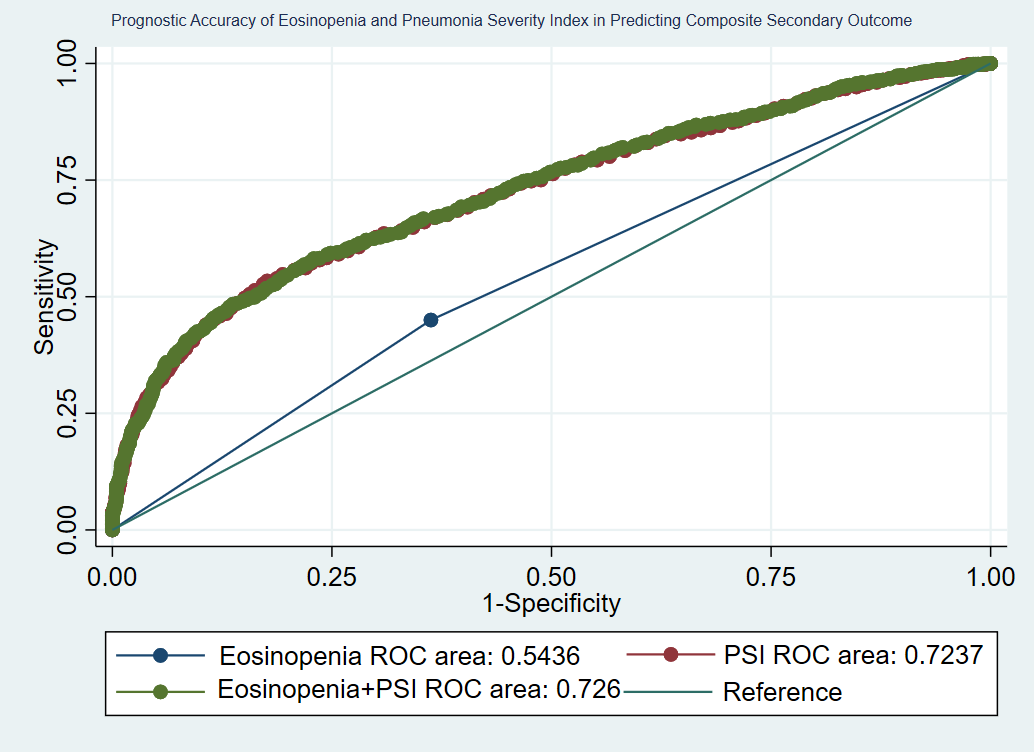


*PSI: Pneumonia severity index
**Compiste outcome: Intensive care unit admission, need for Invasive Ventilation or Non-Invasive Ventilation or vasopressor support

| **Outcomes** | **Eosinopenia  (n=223)** | **No-eosinopenia (n=327)** | **Univariate analysis** | **Multivariate analysis** |
| --- | --- | --- | --- | --- |
|  |  |  | Odds ratio (95% CI),Estimate (95% CI) | |
| **Primary Outcome** |  |  |  |  |
| In-hospital death | 7 | 4 | 2.62 (0.76-9.06) | 2.16 (0.68-6.8) |
| 30-day mortality | 10 | 13 | 1.13 (0.49-2.63) | 0.90 (0.400-2.01) |
| **Secondary Outcome** |  |  |  |  |
| Need for NIV | 19 | 24 | 1.21 (0.64-2.27) | 0.92 (0.47-1.79) |
| Invasive Ventilation | 12 | 19 | 0.96 (0.46-2.03) | 0.6 (0.27-1.33) |
| Need for NIV+IMV | 14 | 13 | 1.64 (0.75-3.57) | 1.21(0.52-2.81) |
| ICU admission | 82 | 91 | **1.50 (1.04-2.17)** | 1.21 (0.83-1.79) |
| Vasopressors support | 47 | 67 | 1.04 (0.68-1.58) | 0.77 (0.48-1.21) |
| Length of hospital stay | 4.7(5.6) | 4.1(4.9) | MD:0.50 ( -0.73-1.73) | MD:-0.01 (-1.25-1.24) |

**Table S-1: Clinical Outcomes based on Eosinopenic Status in subgroup of patients with CRP**

*ICU; Intensive care unit, IMV; Invasive Ventilation, NIV; Non-Invasive Ventilation, MD: Mean difference
** Multivariate analysis: adjusted for Pneumonia severity index, COPD, CRP

**Table S-2: Clinical Outcomes based on Eosinopenic Status in subgroup of patients less than 65 years old**

| **Outcomes** | **Eosinopenia  (n=285)** | **No-eosinopenia (n=458)** | **Univariate analysis** | **Multivariate analysis** |
| --- | --- | --- | --- | --- |
|  |  |  | Odds ratio (95% CI),Estimate (95% CI) | |
| **Primary Outcome** |  |  |  |  |
| In-hospital death | 3 | 4 | 1.21 (0.27-5.44) | 0.95 (0.20-4.57) |
| 30-day mortality | 6 | 9 | 1.07 (0.38-3.05) | 0.92 (0.30-2.8) |
| **Secondary Outcomes** |  |  |  |  |
| Need for NIV | 25 | 36 | 1.18 (0.69-2.02) | 1.03 (0.58-1.83) |
| Invasive Ventilation | 30 | 42 | 1.21 (0.74-1.99) | 0.76 (0.43-1.38) |
| Need for NIV+IMV | 20 | 23 | 1.48 (0.79-2.76) | 1.18 (0.59-2.35) |
| ICU admission | 144 | 172 | **1.69 (1.26-2.29)** | **1.53 (1.07-2.18)** |
| Vasopressors support | 66 | 84 | 1.34 (0.93-1.93) | 1.12 (0.75-1.69) |
| Length of hospital stay | 3.8 | 3.5 | MD: 0.59 (-0.49-1.66) | MD:0.13 (-0.92-1.18) |

*ICU; Intensive care unit, IMV; Invasive Ventilation, NIV; Non-Invasive Ventilation, MD: Mean difference
** Multivariate analysis: adjusted for Pneumonia severity index, COPD

**Table S-3:** **Mortality outcomes based on Eosinopenic Status after adjustment for steroid treatment during hospitalization**

| Outcomes | Eosinopenia  (n=1304) | No-eosinopenia (n=1981) | Univariate analysis | ^**^Multivariate analysis |
| --- | --- | --- | --- | --- |
|  |  |  | Odds ratio (95% CI),Estimate (95% CI) | |
| In-hospital death | 40 | 44 | 1.39 (0.90-2.2) | 1.58 (0.98-2.55) |
| 30-day mortality | 76 | 129 | 0.89 (0.66-1.19) | **1.55 (1.16-2.08)** |

ICU; Intensive care unit, IMV; Invasive Ventilation, NIV; Non-Invasive Ventilation, MD: Mean difference

** Multivariate analysis: adjusted for Pneumonia severity index, COPD, steroid use

| **Outcomes** | **Eosinopenia  (n=422)** | **No-eosinopenia (n=642)** | **Univariate analysis** | **Multivariate analysis** | |
| --- | --- | --- | --- | --- | --- |
|  |  |  | Odds ratio (95% CI),Estimate (95% CI) | | |
| In-hospital death | 15 | 26 | 0.87 (0.46-1.67) | | 0.74 (0.38-1.45) |
| 30-day mortality | 25 | 53 | 0.70 (0.43-1.14) | | **0.57 (0.34-0.96)** |

**Table S-4:** **Mortality outcomes based on Eosinopenic Status after adjustment for early (within 24 hours) steroid treatment**

ICU; Intensive care unit, IMV; Invasive Ventilation, NIV; Non-Invasive Ventilation, MD: Mean difference

** Multivariate analysis: adjusted for Pneumonia severity index, COPD, early steroid use
