# Supplementary material for: Eosinopenia as a predictor of clinical outcomes in hospitalized patients with community-acquired pneumonia: A retrospective cohort study
Source: PLoS One. 2025 Mar 6;20(3):e0314336. doi: 10.1371/journal.pone.0314336 (PMC11884692; doi:10.1371/journal.pone.0314336)
Supplement: S1 Table — (DOCX) [file pone.0314336.s004.docx]

| **Outcomes** | **Eosinopenia  (n=223)** | **No-eosinopenia (n=327)** | **Univariate analysis** | **Multivariate analysis** |
| --- | --- | --- | --- | --- |
|  |  |  | Odds ratio (95% CI),Estimate (95% CI) | |
| **Primary Outcome** |  |  |  |  |
| In-hospital death | 7 | 4 | 2.62 (0.76-9.06) | 2.16 (0.68-6.8) |
| 30-day mortality | 10 | 13 | 1.13 (0.49-2.63) | 0.90 (0.400-2.01) |
| **Secondary Outcome** |  |  |  |  |
| Need for NIV | 19 | 24 | 1.21 (0.64-2.27) | 0.92 (0.47-1.79) |
| Invasive Ventilation | 12 | 19 | 0.96 (0.46-2.03) | 0.6 (0.27-1.33) |
| Need for NIV+IMV | 14 | 13 | 1.64 (0.75-3.57) | 1.21(0.52-2.81) |
| ICU admission | 82 | 91 | **1.50 (1.04-2.17)** | 1.21 (0.83-1.79) |
| Vasopressors support | 47 | 67 | 1.04 (0.68-1.58) | 0.77 (0.48-1.21) |
| Length of hospital stay | 4.7(5.6) | 4.1(4.9) | MD:0.50 ( -0.73-1.73) | MD:-0.01 (-1.25-1.24) |

**Table S-1: Clinical Outcomes based on Eosinopenic Status in subgroup of patients with CRP**

*ICU; Intensive care unit, IMV; Invasive Ventilation, NIV; Non-Invasive Ventilation, MD: Mean difference
** Multivariate analysis: adjusted for Pneumonia severity index, COPD, CRP
