# Supplementary material for: Eosinopenia as a predictor of clinical outcomes in hospitalized patients with community-acquired pneumonia: A retrospective cohort study
Source: PLoS One. 2025 Mar 6;20(3):e0314336. doi: 10.1371/journal.pone.0314336 (PMC11884692; doi:10.1371/journal.pone.0314336)
Supplement: S2 Table — (DOCX) [file pone.0314336.s005.docx]

**Table S-2: Clinical Outcomes based on Eosinopenic Status in subgroup of patients less than 65 years old**

| **Outcomes** | **Eosinopenia  (n=285)** | **No-eosinopenia (n=458)** | **Univariate analysis** | **Multivariate analysis** |
| --- | --- | --- | --- | --- |
|  |  |  | Odds ratio (95% CI),Estimate (95% CI) | |
| **Primary Outcome** |  |  |  |  |
| In-hospital death | 3 | 4 | 1.21 (0.27-5.44) | 0.95 (0.20-4.57) |
| 30-day mortality | 6 | 9 | 1.07 (0.38-3.05) | 0.92 (0.30-2.8) |
| **Secondary Outcomes** |  |  |  |  |
| Need for NIV | 25 | 36 | 1.18 (0.69-2.02) | 1.03 (0.58-1.83) |
| Invasive Ventilation | 30 | 42 | 1.21 (0.74-1.99) | 0.76 (0.43-1.38) |
| Need for NIV+IMV | 20 | 23 | 1.48 (0.79-2.76) | 1.18 (0.59-2.35) |
| ICU admission | 144 | 172 | **1.69 (1.26-2.29)** | **1.53 (1.07-2.18)** |
| Vasopressors support | 66 | 84 | 1.34 (0.93-1.93) | 1.12 (0.75-1.69) |
| Length of hospital stay | 3.8 | 3.5 | MD: 0.59 (-0.49-1.66) | MD:0.13 (-0.92-1.18) |

*ICU; Intensive care unit, IMV; Invasive Ventilation, NIV; Non-Invasive Ventilation, MD: Mean difference
** Multivariate analysis: adjusted for Pneumonia severity index, COPD
