# Supplementary material for: Eosinopenia as a predictor of clinical outcomes in hospitalized patients with community-acquired pneumonia: A retrospective cohort study
Source: PLoS One. 2025 Mar 6;20(3):e0314336. doi: 10.1371/journal.pone.0314336 (PMC11884692; doi:10.1371/journal.pone.0314336)
Supplement: S3 Table — (DOCX) [file pone.0314336.s006.docx]

**Table S-3:** **Mortality outcomes based on Eosinopenic Status after adjustment for steroid treatment during hospitalization**

| Outcomes | Eosinopenia  (n=1304) | No-eosinopenia (n=1981) | Univariate analysis | ^**^Multivariate analysis |
| --- | --- | --- | --- | --- |
|  |  |  | Odds ratio (95% CI),Estimate (95% CI) | |
| In-hospital death | 40 | 44 | 1.39 (0.90-2.2) | 1.58 (0.98-2.55) |
| 30-day mortality | 76 | 129 | 0.89 (0.66-1.19) | **1.55 (1.16-2.08)** |

ICU; Intensive care unit, IMV; Invasive Ventilation, NIV; Non-Invasive Ventilation, MD: Mean difference

** Multivariate analysis: adjusted for Pneumonia severity index, COPD, steroid use
