# Supplementary material for: Eosinopenia as a predictor of clinical outcomes in hospitalized patients with community-acquired pneumonia: A retrospective cohort study
Source: PLoS One. 2025 Mar 6;20(3):e0314336. doi: 10.1371/journal.pone.0314336 (PMC11884692; doi:10.1371/journal.pone.0314336)
Supplement: S4 Table — (DOCX) [file pone.0314336.s007.docx]

**Table S-4:** **Mortality outcomes based on Eosinopenic Status after adjustment for early (within 24 hours) steroid treatment**

| **Outcomes** | **Eosinopenia  (n=422)** | **No-eosinopenia (n=642)** | **Univariate analysis** | **Multivariate analysis** | |
| --- | --- | --- | --- | --- | --- |
|  |  |  | Odds ratio (95% CI),Estimate (95% CI) | | |
| In-hospital death | 15 | 26 | 0.87 (0.46-1.67) | | 0.74 (0.38-1.45) |
| 30-day mortality | 25 | 53 | 0.70 (0.43-1.14) | | **0.57 (0.34-0.96)** |

ICU; Intensive care unit, IMV; Invasive Ventilation, NIV; Non-Invasive Ventilation, MD: Mean difference

** Multivariate analysis: adjusted for Pneumonia severity index, COPD, early steroid use
