# Supplementary material for: Eosinopenia as a predictor of clinical outcomes in hospitalized patients with community-acquired pneumonia: A retrospective cohort study
Source: PLoS One. 2025 Mar 6;20(3):e0314336. doi: 10.1371/journal.pone.0314336 (PMC11884692; doi:10.1371/journal.pone.0314336)
Supplement: Attachment — Submitted filename: Response to Reviewers.docx [file pone.0314336.s010.docx]

Dear Dr. Nosheen Nasir

Thank you for the consideration for our paper, we are resubmitting a revised manuscript accounting for all the comments and points that were resided by the reviewer. This cover letter details our response to each of the comments.

Sincerely,

Wigdan Farah MBBS, MPH

statement in your cover letter. Guidelines for resubmitting your figure files are available below the reviewer comments at the end of this letter.

Journal Requirements:

1. When submitting your revision, we need you to address these additional requirements.

Thank you so much for the comments. We reedited the manuscript to meet the additional requirement. Unfortunately, we tried to create LaTeX submission file however we were not able to add citation. Therefore, we submitted doc file.

2. Thank you for stating in your Funding Statement:

Thank you, please find our amended Funding Statement.

“This research was supported by grant number K23HL168212 (National Institute of Health, recipient: YO), Robert D. and Patricia E. Kern Center for the Science of Health Care Delivery, Mayo Clinic (recipient: YO) and Robert A. Winn Diversity in Clinical Trials Career Development Award (recipient: YO). There was no additional internal or external funding received for this study”

3. We note that you have indicated that there are restrictions to data sharing for this study. For studies involving human research participant data or other sensitive data, we encourage authors to share de-identified or anonymized data. However, when data cannot be publicly shared for ethical reasons, we allow authors to make their data sets available upon request. For information on unacceptable data access restrictions, please see <http://journals.plos.org/plosone/s/data-availability#loc-unacceptable-data-access-restrictions>.

Addressed. Data file was submitted as Supporting Information file (S1 File. Dataset). The data Availability statement in the submission form adjusted accordingly.

4. Please review your reference list to ensure that it is complete and correct. If you have cited papers that have been retracted, please include the rationale for doing so in the manuscript text, or remove these references and replace them with relevant current references. Any changes to the reference list should be mentioned in the rebuttal letter that accompanies your revised manuscript. If you need to cite a retracted article, indicate the article’s retracted status in the References list and also include a citation and full reference for the retraction notice.

Addressed, thank you.

**Comments to the Author**

**Reviewer #1:** The study is interesting considering the fact that eosinopenia may be helpful in identifying patients with high risk of mortality and optimising management strategies accordingly. I have certain queries for the authors:
1) What exactly were the criteria for labelling as CAP? Please specify precisely the clinical, laboratory and radiology criteria for defining CAP in your study?

Thank you for your comment., The presence of CAP was determined using International Classification of Diseases 9 (481–486) and 10 (J13, J15, and J18) codes, coupled with note searches of CAP documented in the diagnosis section of notes on the admission day. Line 68-71. Unfortunately, due to the nature of our database, we are unable to identify the specific imaging modalities used to confirm these diagnoses or the detailed radiological findings.

2) Were patients who were on antihistamines excluded from the study as that could have interfered with the eosinophil count?

Thank you for the comment we agree this a great point however given the limitation of our data we edited our study limitation to highlight this. “Line 217-218”

3) What proportion of the patients were referred from the EMD and how many were admitted from the OPD?

All of the patients were admitted through the emergency department.

4) Was there a correlation between the two groups and the D dimer, pro calcitonin , pro BNP, baseline PaCO2 levels?

Thank you so much for your comment. we redid the analysis and adjusted the manuscript to address this. Please see Table 1 and line 129-130.

5) What were the first line antibiotics used in the patients and was there any specific antibiotic policy adopted for the patients?

Thank you so much for the comment. All patients were treated with guideline-recommended antibiotics based on the institutional pneumonia order sets. However, information about specific antibiotics was not collected in this dataset. “Line71-73”

6) Was there a seasonal variation in the pattern of CAP observed in the patients?

Thank you so much for your comment. The monthly admission trends showed a higher rate of CAP admission during the fall and winter months with reduction in admission rate during the summer months. Similar data was also noted when evaluating the early trends

admission trends by month

Admission count

|  | Total |
| --- | --- |
| January | 321 |
| February | 304 |
| March | 340 |
| April | 307 |
| May | 279 |
| June | 220 |
| July | 191 |
| August | 212 |
| September | 225 |
| October | 283 |
| November | 272 |
| December | 331 |

admission trends by month

Admission count

|  | Total |
| --- | --- |
| January 2009 | 26 |
| January 2010 | 22 |
| January 2011 | 21 |
| January 2012 | 19 |
| January 2013 | 39 |
| January 2014 | 22 |
| January 2015 | 40 |
| January 2016 | 26 |
| January 2017 | 28 |
| January 2018 | 48 |
| January 2019 | 30 |
| February 2009 | 26 |
| February 2010 | 28 |
| February 2011 | 20 |
| February 2012 | 28 |
| February 2013 | 31 |
| February 2014 | 20 |
| February 2015 | 30 |
| February 2016 | 24 |
| February 2017 | 37 |
| February 2018 | 34 |
| February 2019 | 26 |
| March 2009 | 24 |
| March 2010 | 32 |
| March 2011 | 32 |
| March 2012 | 29 |
| March 2013 | 24 |
| March 2014 | 25 |
| March 2015 | 31 |
| March 2016 | 36 |
| March 2017 | 45 |
| March 2018 | 41 |
| March 2019 | 21 |
| April 2009 | 24 |
| April 2010 | 28 |
| April 2011 | 21 |
| April 2012 | 20 |
| April 2013 | 17 |
| April 2014 | 31 |
| April 2015 | 23 |
| April 2016 | 33 |
| April 2017 | 35 |
| April 2018 | 49 |
| April 2019 | 26 |
| May 2009 | 17 |
| May 2010 | 24 |
| May 2011 | 26 |
| May 2012 | 25 |
| May 2013 | 28 |
| May 2014 | 37 |
| May 2015 | 25 |
| May 2016 | 24 |
| May 2017 | 34 |
| May 2018 | 21 |
| May 2019 | 18 |
| June 2009 | 20 |
| June 2010 | 17 |
| June 2011 | 15 |
| June 2012 | 14 |
| June 2013 | 23 |
| June 2014 | 23 |
| June 2015 | 21 |
| June 2016 | 23 |
| June 2017 | 20 |
| June 2018 | 22 |
| June 2019 | 22 |
| July 2009 | 14 |
| July 2010 | 18 |
| July 2011 | 19 |
| July 2012 | 16 |
| July 2013 | 16 |
| July 2014 | 19 |
| July 2015 | 26 |
| July 2016 | 15 |
| July 2017 | 19 |
| July 2018 | 11 |
| July 2019 | 18 |
| August 2009 | 14 |
| August 2010 | 10 |
| August 2011 | 6 |
| August 2012 | 25 |
| August 2013 | 24 |
| August 2014 | 20 |
| August 2015 | 29 |
| August 2016 | 21 |
| August 2017 | 15 |
| August 2018 | 18 |
| August 2019 | 30 |
| September 2009 | 27 |
| September 2010 | 18 |
| September 2011 | 16 |
| September 2012 | 21 |
| September 2013 | 23 |
| September 2014 | 22 |
| September 2015 | 25 |
| September 2016 | 23 |
| September 2017 | 12 |
| September 2018 | 15 |
| September 2019 | 23 |
| October 2009 | 26 |
| October 2010 | 19 |
| October 2011 | 24 |
| October 2012 | 22 |
| October 2013 | 26 |
| October 2014 | 22 |
| October 2015 | 39 |
| October 2016 | 28 |
| October 2017 | 31 |
| October 2018 | 23 |
| October 2019 | 23 |
| November 2009 | 11 |
| November 2010 | 17 |
| November 2011 | 22 |
| November 2012 | 32 |
| November 2013 | 18 |
| November 2014 | 23 |
| November 2015 | 21 |
| November 2016 | 27 |
| November 2017 | 34 |
| November 2018 | 29 |
| November 2019 | 38 |
| December 2009 | 15 |
| December 2010 | 24 |
| December 2011 | 21 |
| December 2012 | 19 |
| December 2013 | 24 |
| December 2014 | 49 |
| December 2015 | 35 |
| December 2016 | 39 |
| December 2017 | 44 |
| December 2018 | 33 |
| December 2019 | 28 |

7) Was history of vaccination like pneumococcal or influenza taken into account as these could have a protective role in CAP?

Thank you for the comment we agree this a great point unfortunately given the limitation of our data we are not able to assess this information. We edited our study limitation to highlight this. “Line 216-217”

8) Line no. 137 seems to be incomplete.
thank you for the comment, this was adjusted

**Reviewer #2:** Respected author,
The manuscript written , titled as “Eosinopenia as a predictor of clinical outcomes in hospitalized patients with community-acquired pneumonia: a retrospective cohort study” addresses an important issue, addresses an important issue, inflammatory marker as predictor of outcomes in community acquired pneumonia. It highlights that the eosinopenia is a strong predictor of poor outcomes for patients with CAP however varies factors such as COPD and history of steroid intake may influence it.
There were a few concerns raised by the reviewer which may need to be addressed by the authors for better understanding and scientific soundness of the article. As researchers have honestly highlighted, several bias like selection bias, recall bias, missing data , and the unmeasured underlying disease status are a major limitation of the retrospective electronic data collection.
Errors have been noted in the representation of statistical data at various instances. Authors furthermore requested to rectify the various spelling and grammatical errors that have been overlooked. Authors are urged to revise the numbering of the references as per the journals recommendation and as per standard practice.

Thank you for the comment we edited the manuscript to address these concerns


While revising your submission, please upload your figure files to the Preflight Analysis and Conversion Engine (PACE) digital diagnostic tool, [https://pacev2.apexcovantage.com/](https://nam12.safelinks.protection.outlook.com/?url=https%3A%2F%2Fpacev2.apexcovantage.com%2F&data=05%7C02%7CFarah.Wigdan%40mayo.edu%7Cae54051806ca4b6e0bbc08dc8c923e57%7Ca25fff9c3f634fb29a8ad9bdd0321f9a%7C0%7C0%7C638539807974831906%7CUnknown%7CTWFpbGZsb3d8eyJWIjoiMC4wLjAwMDAiLCJQIjoiV2luMzIiLCJBTiI6Ik1haWwiLCJXVCI6Mn0%3D%7C0%7C%7C%7C&sdata=wbWk6cOovNGITFt%2FXJcSg6ZCP3soIl528pVQyZVhJ2w%3D&reserved=0). PACE helps ensure that figures meet PLOS requirements. To use PACE, you must first register as a user. Registration is free. Then, login and navigate to the UPLOAD tab, where you will find detailed instructions on how to use the tool. If you encounter any issues or have any questions when using PACE, please email PLOS at [figures@plos.org](mailto:figures@plos.org). Please note that Supporting Information files do not need this step.
