# Supplementary material for: Eosinopenia as a predictor of clinical outcomes in hospitalized patients with community-acquired pneumonia: A retrospective cohort study
Source: PLoS One. 2025 Mar 6;20(3):e0314336. doi: 10.1371/journal.pone.0314336 (PMC11884692; doi:10.1371/journal.pone.0314336)
Supplement: Attachment — Submitted filename: Review_Plos One.docx [file pone.0314336.s009.docx]

Dear Authors,

Although I believe this article does address important information and adds to the body of knowledge surrounding the association between eosinopenia and clinical outcomes in hospitalized patients with CAP. I had many outlying questions that were not answered, such as:

1. This study attempts to analyze Eosinopenia is an independent predictor of adverse outcomes and a marker of severity in bacterial infections, describing the association between eosinopenia and clinical outcomes in hospitalized patients with CAP and looking for associations with increased risk of mortality in hospitalized patients with community-acquired pneumonia. The authors used study design retrospective analysis of adults hospitalized with CAP from January 2009 to December 2019 at a large academic medical center, with Participants 3,285 patients, about 1,304 (39.70%) were classified as eosinopenia. Data collected from demographics, disease severity, comorbidities, smoking history, inflammatory markers, blood eosinophil levels, mortality, length of hospital stay, ICU admission, and need for mechanical ventilation. Statistical methods used nonparametric Wilcoxon rank sum test for continuous variables, chi-square test for categorical variables, and logistic regression with robust standard errors for outcome associations.

Several previous studies…A study by Mao et al [17] showed an association between eosinopenia and increased risk of long-term mortality in hospitalized patients with CAP but with a concurrent diagnosis of acute exacerbation of COPD. Notably, there was a significant difference in total steroid exposure between the eosinopenic and non-eosinopenic groups in this study. Similar results were also reported in a second study by Echevarria et al [21] which focused on a group of hospitalized patients with a diagnosis of CAP, excluding patients with an underlying diagnosis of chronic respiratory disease in an attempt to address the effects of steroids on eosinophils. Although both studies reported a significant increase in CRP in the eosinopenic group compared with the non-eosinopenic group, indicating a higher disease severity in the eosinopenic group, CRP levels were not considered in the analysis. In addition, both studies were limited by sample size. Conclusion in this study, eosinopenia was not associated with an increased risk of death in hospitalized patients with community-acquired pneumonia. **This is contrary to previously published data and highlights the complexity of CAP prognosis. Can you explain it ?**

2. The authors evaluated the association of eosinopenia in predicting adverse outcomes in 3285 hospitalized patients with CAP. Most (39.70%) patients showed eosinopenia on admission and had higher disease severity scores compared to the non-eosinopenic group. Inflammatory Markers and Disease Severity with Eosinopenic patients having higher C-reactive protein (CRP) levels and higher disease severity scores compared to non-eosinopenic patients. **After adjusting for disease severity, chronic obstructive pulmonary disease (COPD), and CRP, the authors found no significant differences in In-Hospital Mortality, ICU Admission, and Mechanical Ventilation. What is the reason?**
3. The manuscript provides sufficient context for the tables. Table titles appear to describe the content and context. Tables are consistent throughout the manuscript. Consistent color schemes, fonts, and formatting make the manuscript more professional and easy to follow. Tables directly support the conclusions of the manuscript. No Unnecessary Information.
4. The method chosen is the most appropriate to test the hypothesis or answer the research question posed. In this study, the sample size and amount of data collected are sufficient to support the conclusions drawn. **The author does not mention** limitations and potential sources of error in the study, avoiding bias and shortcomings or any factors that may affect the results? So that the methods described are clear and comprehensive enough for other researchers to repeat the study and verify the results.
5. The data presented in the results section directly support the conclusions. A clear relationship between the results and conclusions ensures that the author's interpretation is justified by the data. The conclusions drawn are appropriate to the results and scope of the study.
6. In conclusion, the authors found that patients with eosinopenia on admission were generally sicker, with more severe disease markers and higher levels of inflammation and stress markers. Despite these differences in clinical characteristics, the primary outcomes of in-hospital mortality and 30-day mortality were not significantly different between the eosinopenia and non-eosinopenia groups. However, eosinopenia patients were more likely to require non-invasive and invasive mechanical ventilation and had higher odds of requiring ICU admission and vasopressors, reflecting the need for more severe disease management. Length of hospital stay was longer for eosinopenia patients, although these findings were less consistent after multivariate adjustment.
