# Supplementary material for: Eosinopenia as a predictor of clinical outcomes in hospitalized patients with community-acquired pneumonia: A retrospective cohort study
Source: PLoS One. 2025 Mar 6;20(3):e0314336. doi: 10.1371/journal.pone.0314336 (PMC11884692; doi:10.1371/journal.pone.0314336)
Supplement: Attachment — Submitted filename: Response to Reviewer.docx [file pone.0314336.s011.docx]

Dear Dr. Nosheen Nasir

Thank you for the consideration for our paper, we are resubmitting a revised manuscript accounting for all the comments and points that were resided by the reviewer. This cover letter details our response to each of the comments.

Sincerely,

Wigdan Farah MBBS, MPH

Reviewer #1: All comments except one have been addressed. There was no x ray correlation in this study which I thought was quite important to ascertain the diagnosis and to assess the severity of pneumonia. It would have been nice to know if the eosinopenia was related to the x ray pattern that is unilobar , multilobar pneumonia. Also, it would have been good to know how many viral and bacterial pneumonias were there in the study. I understand it is a retrospective study and the other comments have been included in the discussion section as limitations.

Thank you for your thoughtful feedback. We agree with the reviewer regarding the importance of imaging studies in ascertaining the diagnosis of pneumonia and assessing severity, as well as the potential value in exploring associations between imaging patterns (e.g., unilobar vs. multilobar pneumonia) and blood eosinophil levels. However, given the retrospective nature of this study and the lack of consistent imaging data, we are unable to address this question in the current analysis. We recognize the significance of this aspect, therefore Xray findings are being included in our current prospective observational study in CAP.

Regarding the differentiation between viral and bacterial pneumonia, we acknowledge that this is an important factor. Unfortunately, due to the retrospective design, inconsistencies in microbiological sampling, and the inherent limitations in sputum culture yields, it was challenging to reliably distinguish between bacterial and viral etiologies in this cohort. As discussed in the limitations section, the inability to consistently classify pneumonia etiology is a recognized challenge. We appreciate the reviewer's input and are considering this in our current prospective observational study.

Reviewer #3: The manuscript is improved, but I've still some concern about the main message and the influence of steroid treatment.

It's important to clear that eosinopenia hasn't yet been demonstrated to be an independent risk factor for outcome in community-acquired pneumonia or severe infections. The initial sentence in the Abstract is not true and it should be erased. Also the conclusion of the Abstract should be tuned down in accordance with the results. Please, cite and discuss Lin Y, et al. Silent existence of eosinopenia in sepsis: a systematic review and meta-analysis. BMC Infect Dis. 2021; 21: 471. Actually, eosinopenia was frequently associated to severe infection and sepsis, but it has no shown superiority in comparison with conventional biomarkers.

Also this retrospective study is in accordance with it. Another point is the influence of eosinopenia on steroid treatment results. It is well known that several studies showed a significant positive effect of corticosteroid therapy on mortaly from severe CAP (Confalonieri M, et al. Am J Respir Crit Care Med 2005 and Dequin PF, et al. N Engl J Med 2023). Please, cite both papers and statistically analyze in this retrospective study population if the presence of eosinopenia influences the response to corticosteroid on mortality or other outcomes.

Thank you for highlighting this. We have adjusted our manuscript accordingly. Please see the first sentence in the abstract “Eosinopenia has been reported as a predictor of unfavorable outcomes and a marker of severity in bacterial infections.” and the conclusion “Contrary to previously published data, our analysis didn’t demonstrate an association between eosinopenia and increased mortality risk in hospitalized patients with CAP, highlighting the complexity of CAP prognosis”

Also, we adjusted the manuscript to address the suggested points and added the suggested citation. Please see the result section “Adjusting for steroid treatment showed an increased risk for 30-day mortality in the eosinopenic group compared with the non-eosinopenic group. On the other hand, a lower risk for 30-day mortality was observed after adjusting for early steroid treatment (within 24 hours). (S3 and S4 tables)” and discussion section “Although an increase in 30-day mortality was observed after adjusting for steroid use in the eosinopenic group, a decrease in mortality was noted after adjustment for early steroid use. This suggests a potential benefit of early steroid use (within 24 hours) in severe CAP similar to prior studies, {Dequin, 2023 #39}{Confalonieri, 2005 #38} as compared to late steroid use If this finding is replicated in other studies, eosinopenia could be useful in identifying patients who may benefit from early steroid use.

Reviewer #4: Dear Authors,
Although I believe this article does address important information and adds to the body of knowledge surrounding the association between eosinopenia and clinical outcomes in hospitalized patients with CAP. I had many outlying questions that were not answered, such as:


1. This study attempts to analyze Eosinopenia is an independent predictor of adverse outcomes and a marker of severity in bacterial infections, describing the association between eosinopenia and clinical outcomes in hospitalized patients with CAP and looking for associations with increased risk of mortality in hospitalized patients with community-acquired pneumonia. The authors used study design retrospective analysis of adults hospitalized with CAP from January 2009 to December 2019 at a large academic medical center, with Participants 3,285 patients, about 1,304 (39.70%) were classified as eosinopenia. Data collected from demographics, disease severity, comorbidities, smoking history, inflammatory markers, blood eosinophil levels, mortality, length of hospital stay, ICU admission, and need for mechanical ventilation. Statistical methods used nonparametric Wilcoxon rank sum test for continuous variables, chi-square test for categorical variables, and logistic regression with robust standard errors for outcome associations.
Several previous studies…A study by Mao et al [17] showed an association between eosinopenia and increased risk of long-term mortality in hospitalized patients with CAP but with a concurrent diagnosis of acute exacerbation of COPD. Notably, there was a significant difference in total steroid exposure between the eosinopenic and non-eosinopenic groups in this study. Similar results were also reported in a second study by Echevarria et al [21] which focused on a group of hospitalized patients with a diagnosis of CAP, excluding patients with an underlying diagnosis of chronic respiratory disease in an attempt to address the effects of steroids on eosinophils. Although both studies reported a significant increase in CRP in the eosinopenic group compared with the non-eosinopenic group, indicating a higher disease severity in the eosinopenic group, CRP levels were not considered in the analysis. In addition, both studies were limited by sample size. Conclusion in this study, eosinopenia was not associated with an increased risk of death in hospitalized patients with community-acquired pneumonia. This is contrary to previously published data and highlights the complexity of CAP prognosis. Can you explain it ?

Thank you for the comment. Our goal was to utilize a larger CAP cohort to add on to the existing body of knowledge on the prognostic value of eosinopenia in CAP regardless of a concurrent diagnosis of COPD, We have built on the evidence of the prior studies by Mao et al and Echevarria by including patients with COPD ( similar to Mao et al) and without underlying COPD (similar to Echevarria), which is a more accurate representation of population at risk of CAP. In addition, we have considered steroid treatment by excluding patients with steroid use in the hospital just prior to blood draw for labs to decrease the effect of steroids on eosinophils and adjusted for steroid treatment in a sensitivity analysis. CRP is an inflammatory marker with reportedly high levels associated with worse clinical outcomes in CAP. Has both prior studies and our study reported a significant higher CRP level in the eosinopenic group compared to the non-eosinopenic group, it was important to consider CRP as a potential confounder of mortality and other outcomes. We therefore performed a subgroup analysis in patients with CRP available (previously not done by Mao et al and Echevarria et al). The results of this large CAP cohort study showed no association of eosinopenia with outcomes including mortality even in the subgroup of patients with available CRP therefore highlighting the complexity of prognostication in CAP.

2. The authors evaluated the association of eosinopenia in predicting adverse outcomes in 3285 hospitalized patients with CAP. Most (39.70%) patients showed eosinopenia on admission and had higher disease severity scores compared to the non-eosinopenic group. Inflammatory Markers and Disease Severity with Eosinopenic patients having higher C-reactive protein (CRP) levels and higher disease severity scores compared to non-eosinopenic patients. After adjusting for disease severity, chronic obstructive pulmonary disease (COPD), and CRP, the authors found no significant differences in In-Hospital Mortality, ICU Admission, and Mechanical Ventilation. What is the reason?

Thank you for the comment; the differences in outcomes noted in the subgroup suggest a potential interplay between eosinopenia and other inflammatory markers in influencing disease progression and outcomes, which might reflect a direct effect modification of these factors on the relationship between eosinopenia and clinical outcomes. This is potentially due to the absence or weak association between eosinopenia and adverse clinical outcomes and/or the presence of other confounders highlighting the complexity of CAP prognostication. However, this finding need to be evaluated propectively.

3. The manuscript provides sufficient context for the tables. Table titles appear to describe the content and context. Tables are consistent throughout the manuscript. Consistent color schemes, fonts, and formatting make the manuscript more professional and easy to follow. Tables directly support the conclusions of the manuscript. No Unnecessary Information.

Thank you!


4. The method chosen is the most appropriate to test the hypothesis or answer the research question posed. In this study, the sample size and amount of data collected are sufficient to support the conclusions drawn. The author does not mention limitations and potential sources of error in the study, avoiding bias and shortcomings or any factors that may affect the results? So that the methods described are clear and comprehensive enough for other researchers to repeat the study and verify the results.

Thank you so much for the comment, as highlighted in the discussion section some of the major limitations of our studies are driven from the retrospective nature of the study design “As is common in retrospective observational studies, one of our study's limitations was the utilization of electronic health records to obtain data which may have introduced potential biases due to incomplete or missing variables and the inability to account for unmeasured confounders. The study design could not assess for the influence of unmeasured confounders, such as specific microbial etiologies, individual variations in immune responses, vaccination history, concomitant use of antihistamines agents, prior steroid use before admission or medication effects although the larger sample size of our cohort would have help reduce the impact of this limitation. Furthermore, the absence of mechanistic investigations also restricts our understanding of the underlying pathophysiological mechanisms linking eosinopenia to CAP outcomes”

5. The data presented in the results section directly support the conclusions. A clear relationship between the results and conclusions ensures that the author's interpretation is justified by the data. The conclusions drawn are appropriate to the results and scope of the study.

Thank you!

6. In conclusion, the authors found that patients with eosinopenia on admission were generally sicker, with more severe disease markers and higher levels of inflammation and stress markers. Despite these differences in clinical characteristics, the primary outcomes of in-hospital mortality and 30-day mortality were not significantly different between the eosinopenia and non-eosinopenia groups. However, eosinopenia patients were more likely to require non-invasive and invasive mechanical ventilation and had higher odds of requiring ICU admission and vasopressors, reflecting the need for more severe disease management. Length of hospital stay was longer for eosinopenia patients, although these findings were less consistent after multivariate adjustment.

Thank you so much!
